# Progression of mitral and tricuspid regurgitation in patients with and without atrial fibrillation

**DOI:** 10.3389/fcvm.2026.1878401

**Published:** 2026-07-01

**Authors:** Michael Koechlin, Rebecca E. Paladini, Stefanie Aeschbacher, Christine S. Zuern, Ivo Strebel, Michael Coslovsky, Matea Liskij, Ruben Kerler, Philipp Krisai, Leo H. Bonati, David Conen, Felix Mahfoud, Stefan Osswald, Michael Kühne, Beat A. Kaufmann

**Affiliations:** 1 Department of Cardiology, University Hospital Basel, University of Basel, Basel, Switzerland; 2 Cardiovascular Research Institute Basel, University Hospital Basel, University of Basel, Basel, Switzerland; 3 Department of Clinical Research, University of Basel, University Hospital Basel, Basel, Switzerland; 4 Rheinfelden Rehabilitation Clinic, Rheinfelden, Switzerland; 5 Population Health Research Institute, Hamilton, ON, Canada

**Keywords:** atrial fibrillation, atrioventricular regurgitation, mitral regurgitation, tricuspid regurgitation, valvular disease

## Abstract

**Background:**

Mitral (MR) and tricuspid regurgitation (TR) are common valvular disorders that may progress over time. Atrial fibrillation (AF) affects atrial structure and may contribute to the progression of MR or TR. However, longitudinal data quantifying progression rates are limited. We aimed to assess MR and TR progression in patients with and without AF.

**Methods:**

A total of 412 patients with and without AF from the Swiss-AF and BEAT-AF cohorts between 2010 and 2023 were studied. MR and TR severity were graded using the first and last available transthoracic echocardiogram (TTE). The primary endpoint was any progression. Clinically relevant progression additionally required at least moderate severity on the second TTE. Multivariable logistic regression analyses were performed to examine the association between AF and progression of MR or TR.

**Results:**

Median age at first TTE was 74 years (IQR: 69–81, 26% female). Incidence rates of any MR progression were 7.3 vs. 15.0 and of clinically relevant progression 3.3 vs. 3.9 per 100 person-years in AF vs. non-AF patients. For TR, incidence rates were 9.9 vs. 8.9 and 5.2 vs. 2.1 per 100 person-years, respectively. In multivariable analyses, AF was independently associated with both a higher likelihood of any TR progression and clinically relevant TR progression, but not with MR progression.

**Conclusion:**

AF was associated with higher rates of progression of TR severity, including clinically relevant progression. However, AF was not associated with higher rates of progression of MR.

**Clinical Trial Registration:** clinicaltrial.gov, identifier NCT02105844.

## Graphical Abstract

**Figure:**
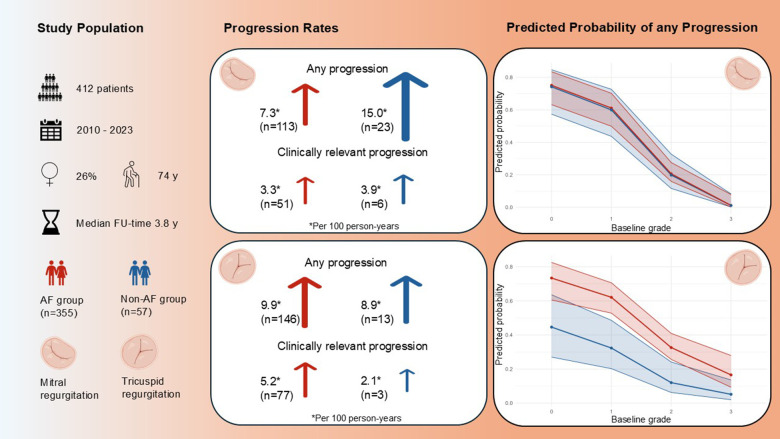
Central illustration; progression rates and predicted probabilities of progression for MR and TR. Figures were created using elements from Biorender.com.

## 1 Introduction

Mitral (MR) and tricuspid (TR) regurgitation are among the most prevalent valvular heart diseases worldwide. The global prevalence of primary MR is estimated to affect up to 24.2 million people, while robust epidemiological data on the prevalence of secondary MR worldwide remain scarce (1–3). In Europe, clinically significant MR is present in up to 5% of the adult population. Of these, approximately 55% have primary (degenerative) MR, 30% have secondary (functional) MR, and the remainder exhibit a combination of both etiologies (2, 4). Likewise, TR is also common, with moderate or severe TR affecting an estimated 1.6 million people in the US (1, 5). Notably, up to 90% of TR cases are secondary resulting from right ventricular dilation and dysfunction (6).

Atrial Fibrillation (AF) is increasingly acknowledged not only as a consequence but also as a potential contributor to atrioventricular valve regurgitation (7, 8). Mechanistically, AF induces structural remodeling of the atria and annulus, leading to leaflet malcoaptation and subsequent regurgitation. This process exhibits distinct pathophysiological characteristics compared to ventricular functional regurgitation (7–9).

Traditionally, functional MR and TR have been primarily ascribed to ventricular dilation and dysfunction, resulting in annular enlargement and leaflet tethering (ventricular functional MR/TR). More recently, a distinct subtype termed atrial functional regurgitation (AFMR/AFTR) has been introduced. AFMR/AFTR is characterized by significant regurgitation occurring in the context of persistent AF and atrial enlargement, despite preserved ventricular size and function (7, 8, 10–12). Indeed, AF has been independently associated with a more than threefold increased risk of developing AFMR (13).

Progression rates of valve regurgitation vary by etiology. In AFMR, regression (3.9 per 100 person-years) appears more frequent than progression (1.9 per 100 person-years), while in patients with mitral valve prolapse, a primary MR subtype, progression was estimated at a rate of 5.5 per 100 person-years (14, 15). Regarding TR, regardless of cause, longitudinal observations suggest a dynamic natural history, characterized by both progression and regression. Patients with at least moderate TR showed a progression rate of 10% and a regression rate of 37% over a one year follow-up (16).

Despite growing recognition of AFMR, systematic data on the progression of MR and TR in patients with AF remain limited, especially compared to non-AF patients and in the context of atrial and ventricular remodeling and functional indices. This study aimed to investigate the natural history of atrioventricular regurgitation in patients with compared to patients without AF, using serial echocardiographic follow-up from two large prospective cohorts.

## 2 Methods

### 2.1 Patient population

We included patients with and without AF from two ongoing prospective multicenter cohort studies in Switzerland: the Swiss Atrial Fibrillation (Swiss-AF, *n* = 3,418), and Basel Atrial Fibrillation (BEAT-AF, *n* = 1,526) studies.

Swiss-AF enrolled a total of 2,415 patients with AF between 2014 and 2017. Patients were eligible if they had documented AF and were ≥65 years of age. A small group (10%) of patients <65 years was enrolled to analyze the effects of AF on individuals in the active workforce. Exclusion criteria were short and reversible forms of AF (e.g., after cardiac surgery or severe sepsis), any acute illness within the preceding 4 weeks, and inability to provide informed consent.

In addition to the AF group, Swiss-AF also enrolled a non-AF group encompassing a total of 1,003 patients between 2018 and 2023. Patients had to be ≥65 years of age, have no history of AF and show sinus rhythm on the ECG at enrolment. Patients were excluded if they had a history of AF, atrial flutter or other relevant supraventricular arrhythmias, dementia (suspected or diagnosed), contraindications for brain MRIs, or an inability to give informed consent.

BEAT-AF enrolled 1,545 patients with AF from 2010 to 2014 across 9 centers in Switzerland. Inclusion and exclusion criteria were nearly identical to Swiss-AF, yet there was no age restriction. Detailed descriptions of both study designs have been reported previously (ClinicalTrial.gov Identifier: NCT02105844) (17, 18).

In this analysis, only patients who participated at the University Hospital of Basel (Swiss-AF: patients with AF *n* = 646, patients without AF *n* = 466, BEAT AF *n* = 1,228) were included. Co-enrollment was not possible and access to clinical data and TTE was obtained in the context of the ongoing cohort studies.

Inclusion required at least two transthoracic echocardiograms (TTE) performed at the University Hospital Basel with a minimum of 180 days between the first and last TTE. Patients were excluded if no TTE data were available, or if the interval between the closest cohort study visit and the first TTE exceeded one year to ensure temporal alignment of clinical and echocardiographic data. In addition, patients with previous mitral or tricuspid valve surgery were excluded. For progression analyses, patients with an already severe mitral- or tricuspid regurgitation were excluded to avoid ceiling effects.

The study complies with the Declaration of Helsinki. Approval by local research ethics committees was obtained before initiation and written informed consent was provided by all study participants.

### 2.2 Data sources

#### 2.2.1 Clinical data

The Swiss-AF and BEAT-AF databases include comprehensive information on patient characteristics, medical history, cardiovascular risk factors, comorbidities, medications, and details of AF diagnosis and management, as described previously (17, 18). Visits were conducted yearly. For each patient, the clinical parameters were extracted from the study visit (either baseline or follow-up) that was closest in time to the date of the first available TTE.

#### 2.2.2 Echocardiographic data

Echocardiographic data were retrieved from the institutional digital echocardiography archive. Detailed measurements of mitral and tricuspid valve function and morphology, as well as chamber dimensions and functional parameters (including left atrial volume index, left ventricular end-diastolic volume index, and left ventricular ejection fraction), were extracted according to current echocardiographic guidelines. Where multiple TTEs were available, the earliest and latest examinations were manually selected for analysis. In cases where key echocardiographic parameters were missing, retrospective, unblinded manual review of TTE images was performed by a trained cardiologist and whenever possible, measurements were added to minimize missing data. For a subgroup analysis including patients with moderate MR, etiology was determined from the echocardiographic report, and where not clearly specified, images were re-reviewed to ensure correct classification.

### 2.3 Endpoints

The major endpoints of this study were the progression of MR and TR over time, as assessed by transthoracic echocardiography. Progression was defined as the change in MR or TR severity grades, respectively, between the first and last available TTE for each patient. Both MR and TR were classified into four grades according to established echocardiographic guidelines: trivial, mild, moderate, and severe (19, 20). For dichotomous analyses, progression was defined as the presence/absence of any increase in severity (≥1 grade), representing the natural course of disease and subsequently as the presence/absence of clinically relevant progression, defined as an increase of at least one grade with at least moderate severity at follow-up.

Secondary endpoints encompassed the change in selected echocardiographic parameters, specifically the left atrial volume index (LAVi), left ventricular end-diastolic volume index (LVEDVi), and left ventricular ejection fraction (LVEF). The progression for each parameter was calculated as the difference between the values at the first and last TTE.

### 2.4 Statistical analysis

Continuous variables are presented as mean ± standard deviation or as median with interquartile range, as appropriate, and compared using Student's *t*-test for normally distributed variables or Wilcoxon rank-sum test for skewed distributions. Categorical variables are summarized as counts and percentages and compared using the chi-squared test or the Fisher's exact test. Echocardiographic characteristics are presented as median values alongside the interquartile range for continuous variables and as counts with percentages for categorical variables. Inter-observer variability was assessed in a blinded fashion in a randomly selected sample of 20 patients using Cohen's kappa statistics.

Group comparisons were performed using the Wilcoxon rank-sum test for continuous data and the chi-squared test or Fisher's exact test for categorical data.

The primary analysis examined the association between AF status and MR and TR progression, assessed as two different dichotomous outcomes. Incidence rates of progression were calculated per 100 person-years (PY). While these rates provide a descriptive measure that facilitates comparison with the existing literature, the exact timing of progression was not available.

Multivariable logistic regression models were then used to identify independent predictors of MR and TR progression, with AF status as the main independent variable. All models included time between the two measurements to account for the different observation times. Model 1 was adjusted for age and sex; Model 2 additionally adjusted for hypertension, coronary artery disease, diabetes mellitus, previous heart failure, and the corresponding baseline regurgitation grade. Baseline MR and TR grades were treated as ordered variables using orthogonal polynomial contrasts, allowing for non-linear trends across increasing baseline regurgitation severity. Odds ratios (ORs) with 95% confidence intervals were derived for all non-valvular covariates. Model results with baseline adjustment were visualized with predicted probabilities averaged over the empirical covariate distribution.

For the secondary endpoints, absolute changes in LAVi, LVEDVi and LVEF were analyzed. Unadjusted mean values with 95% confidence intervals were summarized.

Subsequently, linear regression models were applied to examine associations between AF status and the absolute change of these parameters. The same hierarchical adjustment as in the primary analyses was used, with Model 2 additionally incorporating the respective baseline values of LVEF, LAVi and LVEDVi.

All statistical analyses were conducted using R version 4.4.2 (R Foundation for Statistical Computing, Vienna, Austria). A two-sided *p*-value < 0.05 was considered statistically significant, no correction was done for multiple testing.

## 3 Results

**Figure 1:**
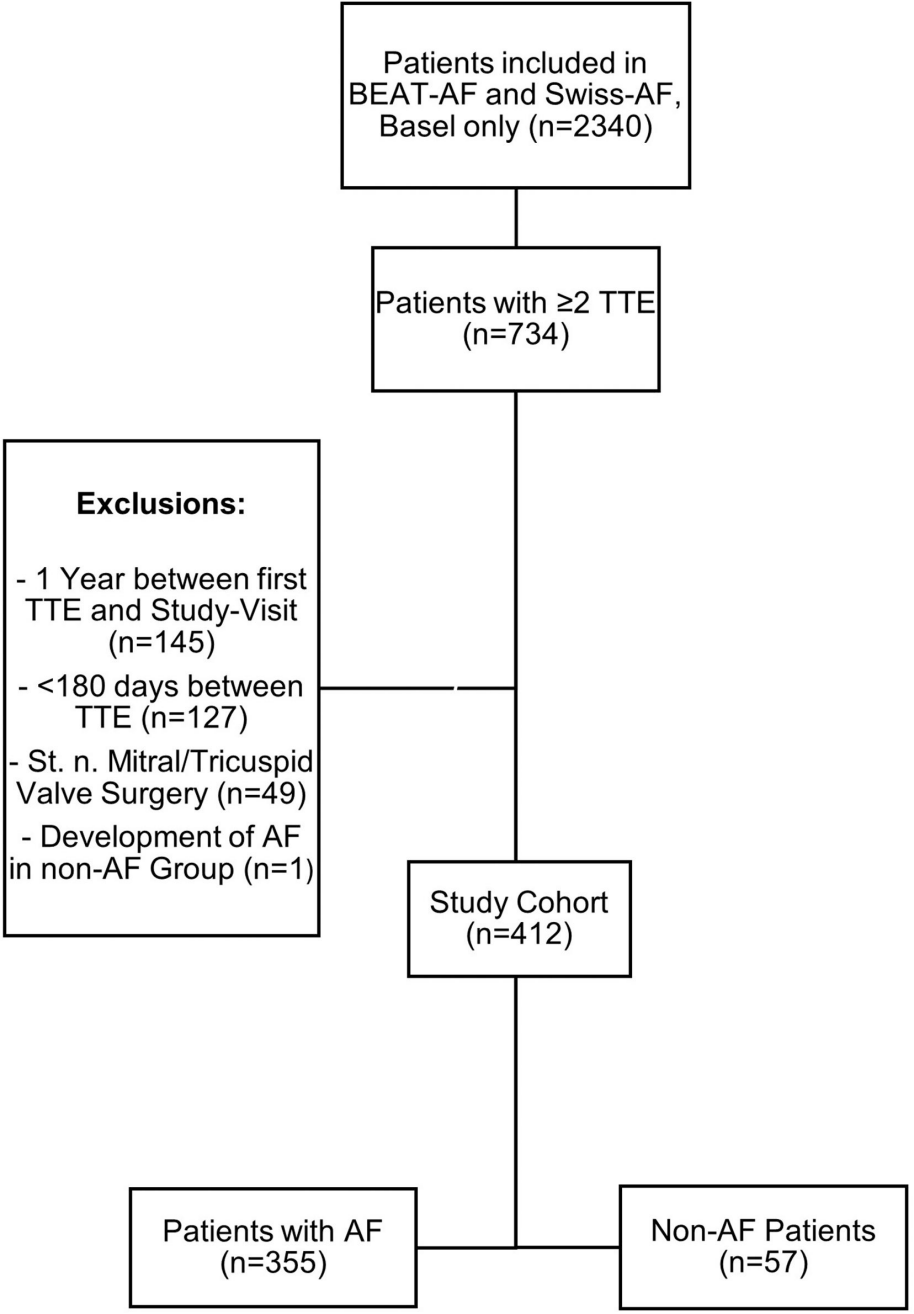
Flowchart of patient selection for the study. A total of 734 patients with ≥2 TTE were identified from the BEAT-AF and Swiss-AF cohort studies. Only patients from Basel were selected.

Of the 2,340 patients enrolled at the University Hospital Basel, 734 had at least two TTE. We excluded 145 patients with >1 year between the first available TTE and a study visit (baseline or follow-up), 127 with <180 days between the two TTE, 49 with prior atrioventricular valve surgery, and one patient without AF who developed AF during follow-up, leaving 412 patients for the current analysis with a median follow-up time of 3.85 years (IQR: 2.10–6.23) (Figure 1).

### 3.1 Clinical baseline characteristics

Clinical baseline characteristics are shown in Table 1. Baseline characteristics were generally well balanced, with a significantly higher percentage of hypertension, previous heart failure, and history of major bleedings in the AF group. Medication use differed substantially between groups, with patients in the AF group more frequently receiving oral anticoagulation and beta-blockers. Antiplatelet therapy was more common in the no AF group.

**Table 1:** Baseline characteristics, stratified by AF status and by AF subtype.

Variable	Overall (*n* = 412)	Control (non-AF) (*n* = 57)	AF (*n* = 355)	*p-value*	Non-paroxysmal AF (*n* = 190)	Paroxysmal AF (*n* = 165)	*p*-value
Sex (Female)	109 (26%)	20 (35%)	89 (25%)	0.111	34 (18%)	55 (33%)	<0.001
Age at first TTE, years	74 [69–81]	73 [69–78]	74 [69–81]	0.457	75 [69–82]	72 [69–81]	0.091
Age at last TTE, years	79 [73–85]	76 [71–81]	79 [74–85]	0.012	81 [75–86]	77 [73–84]	0.020
Hypertension	315 (76%)	35 (61%)	280 (79%)	0.004	154 (81%)	126 (76%)	0.280
Diabetes mellitus	85 (21%)	14 (25%)	71 (20%)	0.430	41 (22%)	30 (18%)	0.425
Smoking status				0.496			0.398
Active	23 (8%)	2 (4%)	21 (8%)		10 (7%)	11 (10%)	
Former	165 (54%)	33 (58%)	132 (53%)		81 (56%)	51 (49%)	
Never smoked	118 (39%)	22 (39%)	96 (39%)		53 (37%)	43 (41%)	
Coronary artery disease	140 (34%)	23 (40%)	117 (33%)	0.274	73 (38%)	44 (27%)	0.019
History of stroke or TIA	74 (18%)	8 (14%)	66 (19%)	0.405	32 (17%)	34 (21%)	0.363
History of myocardial infarction	89 (22%)	16 (28%)	73 (21%)	0.201	45 (24%)	28 (17%)	0.118
History of heart failure	136 (33%)	12 (21%)	124 (35%)	0.039	84 (44%)	40 (24%)	<0.001
History of major bleeding	43 (18%)	4 (7%)	39 (21%)	0.014	20 (20%)	19 (23%)	0.691
Oral anticoagulant therapy	334 (81%)	7 (12%)	327 (92%)	<0.001	184 (97%)	143 (87%)	<0.001
Antiplatelet therapy	94 (23%)	38 (67%)	56 (16%)	<0.001	23 (12%)	33 (20%)	0.042
Betablocker therapy	291 (71%)	25 (44%)	266 (75%)	<0.001	144 (76%)	122 (74%)	0.688
ACE inhibitor therapy	145 (35%)	24 (42%)	121 (34%)	0.239	70 (37%)	51 (31%)	0.239
Calcium-channel blocker therapy	100 (24%)	19 (33%)	81 (23%)	0.086	45 (24%)	36 (22%)	0.676
Angiotensin II Receptor blockers	143 (35%)	14 (25%)	129 (36%)	0.083	73 (38%)	56 (34%)	0.381
Mineralocorticoid receptor antagonists	68 (17%)	7 (12%)	61 (17%)	0.355	44 (23%)	17 (10%)	0.001

Values are presented as numbers (%) for categorical variables and as median [interquartile range] for continuous variables. Differences between groups were assessed using Pearson's Chi-squared test, Fisher's exact test, or the Wilcoxon rank sum test, as appropriate. AF, atrial fibrillation; TTE, transthoracic echocardiography; ACE, angiotensin converting enzyme.

### 3.2 Echocardiographic baseline characteristics

Detailed data on echocardiographic characteristics is presented in Table 2.

**Table 2:** Echocardiographic parameters at baseline and follow-up, stratified by AF status and AF subtype.

Variable	Overall (*n* = 412)	Control (non-AF) (*n* = 57)	AF (*n* = 355)	*p-*value	Non-paroxysmal AF (*n* = 190)	Paroxysmal AF (*n* = 165)	*p-*value
Time between TTE (days)	1,406 [766–2,274]	834 [642–1,296]	1,496 [826–2,419]	<0.001	1,544 [930–2,622]	1,425 [770–2,186]	0.113
Time between study visit and TTE (days)	−50 [−118–56]	−105 [−166–−61]	−26 [−110–65]	<0.001	−40 [−108–73]	−15 [−110–59]	0.608
Time between AF diagnosis and TTE (days)			1,971 [948–3,906]		2,134 [1,163–4,279]	1,880 [800–3,379]	0.048
Heart rate first (bpm)	70 [62–80]	73 [65–78]	70 [61–82]	0.973	75 [64–87]	69 [60–77]	0.004
Heart rate last (bpm)	70 [61–80]	70 [62–81]	70 [61–80]	0.990	70 [60–80]	70 [64–80]	0.207
Systolic BP first (mmHg)	133 [122–148]	135 [116–154]	133 [123–148]	0.723	131 [120–143]	136 [125–150]	0.015
Systolic BP last (mmHg)	134 [118–151]	133 [122–151]	134 [116–151]	0.355	130 [114–147]	140 [121–155]	0.003
Diastolic BP first (mmHg)	78 [70–88]	78 [71–85]	78 [70–88]	0.996	78 [70–88]	78 [70–88]	0.648
Diastolic BP last (mmHg)	77 [69–86]	79 [70–86]	76 [68–85]	0.462	75 [65–86]	79 [70–85]	0.069
LAD first (mm)	43 [37–48]	36 [30–39]	44 [39–49]	<0.001	46 [41–51]	41 [37–45]	<0.001
LAD last (mm)	43 [38–49]	36 [34–40]	44 [39–50]	<0.001	48 [42–53]	41 [36–47]	<0.001
LAV first (mL)	83 [65–108]	56 [42–74]	86 [69–114]	<0.001	100 [81–124]	73 [62–92]	<0.001
LAV last (mL)	90 [69–118]	62 [52–77]	94 [72–122]	<0.001	108 [87–133]	80 [64–102]	<0.001
LAVi first (mL/m²)	43 [33–54]	30 [23–38]	46 [36–55]	<0.001	50 [41–63]	39 [33–49]	<0.001
LAVi last (mL/m²)	47 [37–61]	33 [27–44]	49 [39–63]	<0.001	57 [46–67]	42 [35–55]	<0.001
LVEDD first (mm)	48 [44–53]	46 [42–50]	49 [44–53]	0.006	49 [45–55]	47 [44–52]	0.003
LVEDD last (mm)	48 [43–53]	46 [42–50]	49 [44–54]	0.007	51 [45–55]	46 [43–51]	<0.001
LVESD first (mm)	33 [28–39]	31 [26–34]	34 [29–39]	0.012	35 [30–42]	31 [27–37]	<0.001
LVESD last (mm)	33 [29–40]	30 [27–34]	34 [29–41]	0.001	36 [31–43]	33 [28–36]	<0.001
LVEDV first (mL)	97 [77–118]	86 [61–103]	99 [78–121]	0.002	99 [80–128]	99 [77–118]	0.279
LVEDV last (mL)	90 [72–119]	79 [61–108]	91 [74–120]	0.041	96 [75–131]	86 [73–110]	0.012
LVEDVi first (mL/m²)	50 [40–60]	46 [34–52]	50 [41–62]	0.011	50 [41–64]	50 [41–60]	0.917
LVEDVi last (mL/m²)	46 [38–61]	42 [34–55]	47 [39–62]	0.038	51 [40–67]	45 [38–56]	0.017
LVEF first (%)	55 [47–61]	58 [55–62]	55 [46–60]	0.013	52 [43–60]	57 [53–62]	<0.001
LVEF last (%)	55 [45–60]	60 [55–61]	55 [44–60]	<0.001	54 [41–60]	55 [45–60]	0.005
MR grade first TTE				<0.001			0.002
0	67 (17%)	14 (25%)	53 (15%)		19 (10%)	34 (21%)	
1	89 (22%)	21 (37%)	68 (20%)		29 (16%)	39 (24%)	
2	174 (43%)	22 (39%)	152 (44%)		95 (52%)	57 (35%)	
3	70 (17%)	0 (0%)	70 (20%)		40 (22%)	30 (19%)	
4	2 (0%)	0 (0%)	2 (1%)		1 (1%)	1 (1%)	
MR grade last TTE				<0.001			0.007
0	60 (15%)	14 (25%)	46 (13%)		15 (8%)	31 (19%)	
1	52 (13%)	15 (26%)	37 (11%)		16 (8%)	21 (13%)	
2	189 (46%)	22 (39%)	167 (47%)		98 (52%)	69 (43%)	
3	104 (25%)	5 (9%)	99 (28%)		59 (31%)	40 (25%)	
4	4 (1%)	1 (2%)	3 (1%)		2 (1%)	1 (1%)	
TR grade first TTE				0.004			<0.001
0	67 (17%)	11 (20%)	56 (17%)		19 (11%)	37 (24%)	
1	119 (31%)	26 (48%)	93 (28%)		42 (24%)	51 (33%)	
2	144 (37%)	16 (30%)	128 (39%)		86 (48%)	42 (27%)	
3	50 (13%)	1 (2%)	49 (15%)		27 (15%)	22 (14%)	
4	6 (2%)	0 (0%)	6 (2%)		4 (2%)	2 (1%)	
TR grade last TTE				<0.001			0.004
0	51 (13%)	11 (19%)	40 (12%)		11 (6%)	29 (18%)	
1	88 (22%)	25 (44%)	63 (18%)		31 (17%)	32 (20%)	
2	146 (36%)	16 (28%)	130 (38%)		78 (42%)	52 (33%)	
3	97 (24%)	5 (9%)	92 (27%)		54 (29%)	38 (24%)	
4	20 (5%)	0 (0%)	20 (6%)		13 (7%)	7 (4%)	

Values are presented as numbers (%) for categorical variables and as median [interquartile range] for continuous variables. Differences between groups were assessed using Pearson's Chi-squared test, Fisher's exact test, or the Wilcoxon rank-sum test, as appropriate. BP, blood pressure; AF, atrial fibrillation; MR, mitral regurgitation; TR, tricuspid regurgitation; TTE, transthoracic echocardiography; LAD, left atrial diameter; LAV, left atrial volume; LAVi, left atrial volume index; LVEDD, left ventricular end-diastolic diameter; LVESD, left ventricular end-systolic diameter; IVS, interventricular septum thickness; PW, posterior wall thickness; LVEDV, left ventricular end-diastolic volume; LVEDVi, left ventricular end-diastolic volume index; LVEF, left ventricular ejection fraction.

Compared with no AF patients, patients with AF had larger left atrial and left ventricular dimensions and volumes. LAV and LAVi were markedly higher in AF both at baseline and at follow-up. Similarly, LVEDV and LVEDVi were higher in AF patients at both time points, while LVEF was lower compared with controls. AF patients exhibited a higher prevalence of moderate or greater MR and TR compared to no AF patients, both at baseline and at follow-up. A higher prevalence of moderate or greater MR and TR was observed in patients with non-paroxysmal AF vs. paroxysmal AF both at baseline and follow up.

### 3.3 Major endpoints

#### 3.3.1 Mitral regurgitation

A total of 341 patients with AF and 57 patients without AF were included in this analysis. Incidence rates (per 100 patient years) for any MR progression were 7.3 for AF and 15.0 for patients without AF. For clinically relevant progression, incidence rates were 3.3 vs. 3.9, respectively. For non-paroxysmal and paroxysmal AF, incidence rates for any progression were 7.5 and 6.9, and 3.8 vs. 2.6 for clinically relevant progression (Supplementary Table S1), respectively. Incidence rates stratified by baseline MR grade are provided in Supplementary Table S2.

**Figure 2:**
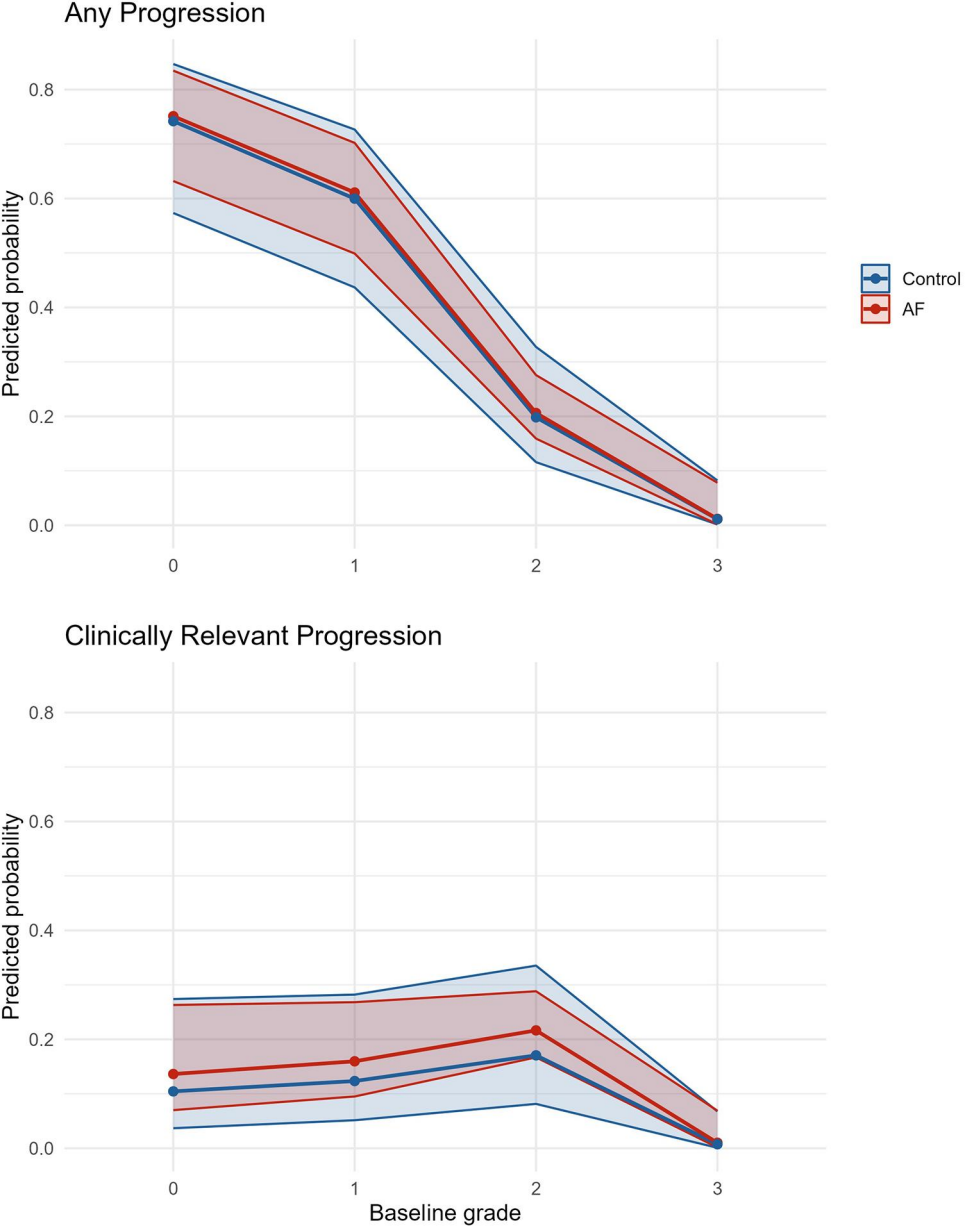
Predicted probability of any and clinically relevant mitral regurgitation progression by baseline grade, stratified by AF status. Model-based probabilities averaged over the empirical distribution of all covariates; shaded areas indicate 95% confidence intervals.

In the multivariable logistic regression model assessing any MR progression, no association was observed for AF, neither for model 1(OR: 0.58, 95% CI: 0.31–1.07, *p* = 0.075), nor for model 2 (OR: 1.05, 95% CI: 0.52–2.18, *p* = 0.885) (Supplementary Table S3). Predicted probabilities for any MR progression by baseline grade are summarized in Supplementary Table S5 and Figure 2. AF was not associated with clinically relevant MR progression (Model 1: OR: 1.02, 95% CI: 0.42–2.87, *p* = 0.97; Model 2: OR: 1.38, 95% CI: 0.54–4.05, *p* = 0.529) (Supplementary Table S4). Predicted probabilities for clinically relevant MR progression by baseline grade are provided in Supplementary Table S6 and Figure 2.

Regarding inter-observer variability for mitral regurgitation grading, exact agreement was 73.7% with an unweighted *κ* of 0.64 and a quadratic weighted *κ* of 0.88, indicating substantial to excellent agreement.

#### 3.3.2 Tricuspid regurgitation

A total of 319 patients with AF and 54 patients without AF were included in this analysis. Incidence rates for any TR progression were 9.9 for patients with AF and 8.9 for patients without AF. For clinically relevant progression, incidence rates were 5.2 vs. 2.1, respectively. Incidence rates for any progression were 9.9 for non-paroxysmal AF and paroxysmal AF, and 6.2 vs. 4.0 for clinically relevant progression (Supplementary Table S1), respectively. Incidence rates stratified by baseline TR grade are provided in Supplementary Table S2.

**Figure 3:**
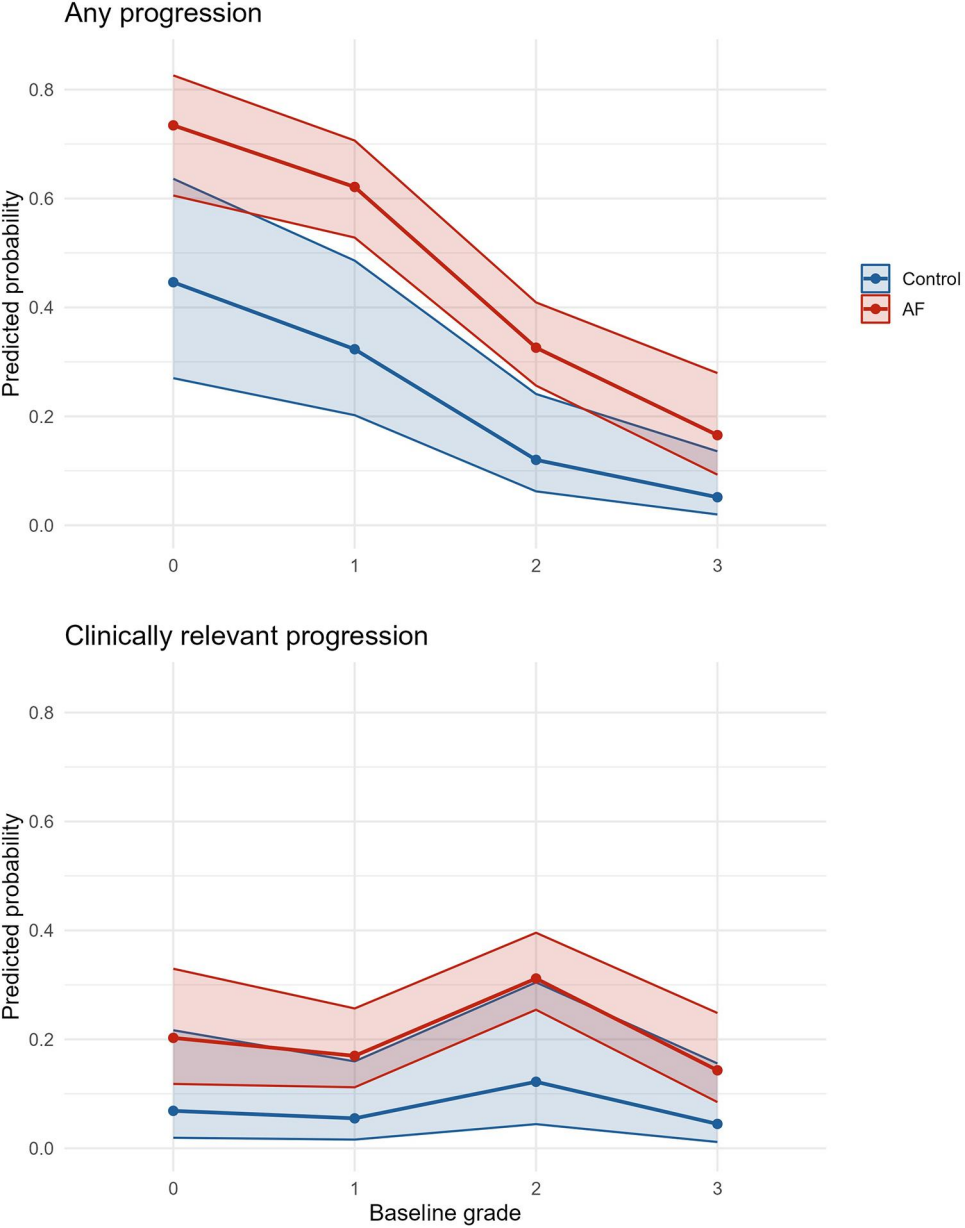
Predicted probability of any and clinically relevant tricuspid regurgitation progression by baseline grade, stratified by AF status. Model-based probabilities averaged over the empirical distribution of all covariates; shaded areas indicate 95% confidence intervals.

In the multivariable logistic regression model, AF was independently associated with increased odds of any TR progression (Model 1: OR: 2.22, 95% CI: 1.15–4.54, *p* = 0.023; Model 2: OR: 3.79, 95% CI: 1.83–8.27, *p* < 0.001) (Supplementary Table S7). Predicted probabilities for any TR progression are provided in Supplementary Table S9 and Figure 3. AF was independently associated with increased odds of clinically relevant TR progression (Model 1: OR: 3.66, 95% CI: 1.22–15.89, *p* = 0.041; Model 2: OR: 3.97, 95% CI: 1.25–17.82, *p* = 0.035) (Supplementary Table S8). Predicted probabilities for clinically relevant TR progression are summarized in Supplementary Table S10 and Figure 3.

For tricuspid regurgitation grading, exact agreement was 84.2% with an unweighted *κ* of 0.80 and a quadratic weighted κ of 0.90, consistent with excellent inter-observer agreement.

### 3.4 Other endpoints

#### 3.4.1 Left atrial volume index

Among 334 patients with paired echocardiographic measurements, the mean LAVI increase was 5.59 mL/m^2^ (95% CI: 3.70–7.47) for the AF group and 4.15 mL/m^2^ (95% CI: 0.79–7.51) for the no AF group. After multivariable adjustment, neither model 1 nor model 2 showed a significant association between AF and change of LAVi (Supplementary Table S11).

#### 3.4.2 Left ventricular end-diastolic volume index

Among 289 patients with paired echocardiographic measurements, the mean LVEDVi change was −0.11 mL/m^2^ (95% CI: −2.46–2.24) for the AF group and −2.12 mL/m^2^ (95% CI: −7.03–2.78) for the no AF group. After multivariable adjustment, neither model 1 nor model 2 showed a significant association between AF and change of LVEDVi (Supplementary Table S12).

#### 3.4.3 Left ventricular ejection fraction

Among 402 patients with paired echocardiographic measurements, the mean absolute LVEF change was −1.49% (95% CI: −2.68–−0.29) for the AF group and 0.32% (95% CI: −2.02–2.67) for the no AF group. After multivariable adjustment, model 1 did not show a significant association between AF and change of LVEF whereas model 2 did (Supplementary Table S13).

### 3.5 Subgroup analysis

To further explore potential differences in progression rates by etiology, a subgroup analysis was performed among patients with moderate MR at baseline within the AF cohort. Progression to severe MR was rare, occurring in only 1 of 70 patients (Supplementary Table S14).

## 4 Discussion

In this study, we evaluated the progression MR and TR, as well as left-sided chamber remodeling, in patients with AF and without AF. Over a median follow-up of 3.85 years, we observed that overall progression of MR and TR was infrequent, particularly for clinically relevant progression. However, distinct patterns emerged: AF was associated with a higher rate of TR progression compared to controls, whereas MR progression did not differ between groups. In the subgroup of AF patients with moderate MR at baseline, progression to severe MR was rare, regardless of MR etiology. Notably, predicted probabilities of progression showed a potential ceiling effect, patients with more advanced baseline regurgitation were less likely to progress further. As anticipated, the highest likelihood of clinically relevant progression was seen in patients with pre-existing mild baseline regurgitation.

Previous studies showed a significant impact of AF on the development and progression of TR, irrespective of other known risk-factors (21, 22). Furthermore, Zhou et al. demonstrated a significantly greater annular dilatation and more severe TR in patients with AF (23). Consistent with these findings, our results also highlight a marked effect of AF on TR. Concerning MR, Naser et al. recently showed that regression (3.9% per year) of MR was more frequent than progression (1.9% per year) in a large cohort (60% with AF), with no association between AF and MR progression. Importantly, that study focused on AFMR and included relatively few progression events (from mild-moderate/moderate to severe) (14). These data suggest, that, despite the expected pathophysiological link between AF, atrial remodeling and progression of atrioventricular valve regurgitation, the actual rate of MR progression may remain low, a finding corroborated by our results.

Secondary or functional atrioventricular regurgitation has classically been attributed to ventricular dilatation and dysfunction. Recently, atrial functional regurgitation has been described as a different entity, characterized by significant regurgitation with absent mitral or tricuspid leaflet disease and preserved ventricular size and function (10, 24). In contrast to ventricular functional regurgitation, atrial functional MR is thought to be primarily driven by atrial remodeling and annular dilatation, frequently in the setting of longstanding AF and heart failure with preserved ejection fraction (25). From this perspective, one would expect AF to be associated with accelerated progression of MR and TR. However, our data only partially support this hypothesis, as a significant effect was observed only for TR.

It should also be noted that the relationship between atrioventricular regurgitation, in particular degenerative and ventricular functional MR, and AF is likely bidirectional (26, 27). In patients under the age of 73 years, even mild MR has been shown to be independently associated with increased AF risk (28). Although we did not observe higher MR progression rates, AF patients herein presented with more advanced degrees of both MR and TR, as well as significantly larger atrial and ventricular volumes and slightly lower left ventricular ejection fraction at both TTE compared to no AF patients. While AF was not associated with greater changes in atrial and ventricular volumes over time, it was linked to a significant decline in LVEF, suggesting deterioration of ventricular function.

These findings indicate that AF is associated with a less favorable atrial and ventricular geometry and function at baseline yet does not necessarily accelerate structural remodeling. They further support the concept of AF-related atrial enlargement being associated with the presence but not necessarily the progression of atrioventricular regurgitation. Interestingly, we also observed that for MR progression in general as well as clinically relevant TR progression, progression rates were higher in non-paroxysmal compared to paroxysmal AF. This suggests that, within AF patients, an expected higher burden of AF may influence the progression rates of atrioventricular regurgitation.

### 4.1 Limitations

This study has several limitations. First, due to its observational character and an overall low number of events, causal inferences regarding AF status and progression of regurgitation cannot be drawn. Furthermore, transthoracic echocardiographic measurements were performed by different sonographers over an extended period, leading to TR grading in only 4 grades as opposed to the 5-grade classification currently employed and may have introduced variability in image acquisition. Reassessment of TTE to reduce missing data was not blinded to AF status, as AF can often be identified from echocardiographic recordings, potentially leading to observer bias. Moreover, the moderate duration of follow-up, the difference in follow-up duration between groups and the small number of control patients due to applied exclusion criteria represent additional limitations, potentially reducing the statistical power of our analyses and limiting the robustness of between-group comparisons. Additionally, a potential selection bias cannot be excluded due to the necessity of a follow-up TTE, which may have preferentially selected patients with more complex cardiovascular conditions. Comprehensive etiologic classification of MR/TR was not consistently available across the entire cohort, particularly in patients with lower-grade regurgitation, limiting etiology-specific analyses. Furthermore, as only the first and the last available TTE were considered for progression analyses, potential intermediate fluctuations in MR/TR severity could not be assessed. Altough time-to-event analyses may be informative in future prospective studies, the non-standardized intervals between echocardiographic examinations in our cohort would have limited the accuracy of event-time estimation. Changes in AF treatment strategy and rhythm status during follow-up were not captured in the present analyses. As these factors may influence cardiac remodeling and valvular regurgitation, their potential impact on progression could not be fully assessed.

## 5 Conclusion

In this longitudinal study, AF was independently associated with higher rates of TR progression, whereas no statistically significant association with MR progression could be demonstrated. Furthermore, AF was associated with larger LAVi, larger LVEDVi and lower LVEF at baseline and follow-up. Importantly, AF was associated with a significant decline in LVEF over time, despite stable chamber sizes. These findings suggest a potential association between AF and ventricular functional decline and support further investigation of AF burden and atrial functional regurgitation as potential therapeutic targets.

## Data Availability

The datasets presented in this article are not readily available because of restrictions by the Ethics Committee. Requests to access the datasets should be directed to the corresponding author.
