# Supplementary material for: Progression of mitral and tricuspid regurgitation in patients with and without atrial fibrillation
Source: Front Cardiovasc Med. 2026 Jul 1;13:1878401. doi: 10.3389/fcvm.2026.1878401 (PMC13369044; doi:10.3389/fcvm.2026.1878401)
Supplement: Supplementary file 1 [file Table1.docx]

**Supplement**

|  | **Group** | **n (missing)** | **Progressions (n)** | **Person-years (PY)** | **Incidence rate (per 100 PY)** | **95% CI** |
| --- | --- | --- | --- | --- | --- | --- |
| **Any MR progression** | AF | 341 (2) | 113 | 1558.3 | **7.3** | 5.98-8.72 |
|  | No AF | 57 (0) | 23 | 153.1 | **15.0** | 9.52-22.50 |
|  | *Non-paroxysmal AF* | 183 (0) | 65 | 865.1 | **7.5** | 5.80-8.58 |
|  | *Paroxysmal AF* | 158 (2) | 48 | 693.2 | **6.9** | 5.11-9.18 |
| **Clinically relevant MR progression** | AF | 341 (2) | 51 | 1558.3 | **3.3** | 2.44-4.30 |
|  | No AF | 57 (0) | 6 | 153.1 | **3.9** | 1.44-8.53 |
|  | *Non-paroxysmal AF* | 183 (0) | 33 | 865.1 | **3.8** | 2.63-5.36 |
|  | *Paroxysmal AF* | 158 (2) | 18 | 693.2 | **2.6** | 1.54-4.10 |
| **Any TR progression** | AF | 319 (7) | 146 | 1473.5 | **9.9** | 8.37-11.70 |
|  | No AF | 54 (0) | 13 | 145.5 | **8.9** | 4.76-15.30 |
|  | *Non-paroxysmal AF* | 171 (3) | 82 | 825.4 | **9.9** | 7.90-12.30 |
|  | *Paroxysmal AF* | 148 (4) | 64 | 648.1 | **9.9** | 7.61-12.60 |
| **Clinically relevant TR progression** | AF | 319 (7) | 77 | 1473.5 | **5.2** | 4.12-6.53 |
|  | No AF | 54 (0) | 3 | 145.5 | **2.1** | 0.43-6.03 |
|  | *Non-paroxysmal AF* | 171 (3) | 51 | 825.4 | **6.2** | 4.60-8.12 |
|  | *Paroxysmal AF* | 148 (4) | 26 | 648.1 | **4.0** | 2.62-5.88 |

**Table S1: Incidence rates of mitral and tricuspid regurgitation progression according to AF status and AF subtype.** Abbreviations: MR = mitral regurgitation; TR = tricuspid regurgitation; AF = atrial fibrillation; PY = person-years; CI = confidence interval

|  | **Baseline grade** | **Group** | **Patients (n)** | **Progressions (n)** | **Person-years (PY)** | **Incidence rate (per 100 PY)** |
| --- | --- | --- | --- | --- | --- | --- |
| **Any MR progression** | None (0) | AF | 53 | 37 | 202.8 | 18.2 |
|  | Trivial (1) | AF | 68 | 41 | 314.9 | 13.0 |
|  | Mild (2) | AF | 152 | 34 | 737.8 | 4.6 |
|  | Moderate (3) | AF | 70 | 1 | 302.8 | 0.3 |
|  | None (0) | No AF | 14 | 10 | 30.8 | 32.4 |
|  | Trivial (1) | No AF | 21 | 9 | 56.7 | 15.9 |
|  | Mild (2) | No AF | 22 | 4 | 65.6 | 6.1 |
| **Clinically relevant MR progression** | None (0) | AF | 53 | 7 | 202.8 | 3.5 |
|  | Trivial (1) | AF | 68 | 9 | 314.9 | 2.9 |
|  | Mild (2) | AF | 152 | 34 | 737.8 | 4.6 |
|  | Moderate (3) | AF | 70 | 1 | 302.8 | 0.3 |
|  | None (0) | No AF | 14 | 0 | 30.8 | 0.0 |
|  | Trivial (1) | No AF | 21 | 2 | 56.7 | 3.5 |
|  | Mild (2) | No AF | 22 | 4 | 65.6 | 6.1 |
| **Any TR progression** | None (0) | AF | 56 | 37 | 237.8 | 15.6 |
|  | Trivial (1) | AF | 93 | 54 | 423.4 | 12.8 |
|  | Mild (2) | AF | 128 | 45 | 614.2 | 7.3 |
|  | Moderate (3) | AF | 49 | 10 | 198.1 | 5.0 |
|  | None (0) | No AF | 11 | 3 | 27.0 | 11.1 |
|  | Trivial (1) | No AF | 26 | 9 | 74.0 | 12.2 |
|  | Mild (2) | No AF | 16 | 1 | 38.9 | 2.6 |
|  | Moderate (3) | No AF | 1 | 0 | 5.6 | 0.0 |
| **Clinically relevant TR progression** | None (0) | AF | 56 | 9 | 237.8 | 3.8 |
|  | Trivial (1) | AF | 93 | 13 | 423.4 | 3.1 |
|  | Mild (2) | AF | 128 | 45 | 614.2 | 7.3 |
|  | Moderate (3) | AF | 49 | 10 | 198.1 | 5.0 |
|  | None (0) | No AF | 11 | 0 | 27.0 | 0.0 |
|  | Trivial (1) | No AF | 26 | 2 | 74.0 | 2.7 |
|  | Mild (2) | No AF | 16 | 1 | 38.9 | 2.6 |
|  | Moderate (3) | No AF | 1 | 0 | 5.6 | 0.0 |

**Table S2:** **Incidence rates of mitral and tricuspid regurgitation progression according to AF status and baseline regurgitation severity.** Abbreviations: AF = atrial fibrillation; MR = mitral regurgitation; TR = tricuspid regurgitation; PY = person-years.

| **Covariate** | **Odds Ratio** | **Lower 95% CI** | **Upper 95% CI** | **p-value** |
| --- | --- | --- | --- | --- |
| **Model 1** | | | | |
| AF (vs no AF) | 0.58 | 0.31 | 1.07 | 0.075 |
| Age at first TTE (y) | 1.00 | 0.98 | 1.03 | 0.867 |
| Male (vs Female) | 1.33 | 0.82 | 2.19 | 0.252 |
| Time between TTE (y) | 1.11 | 1.02 | 1.21 | 0.021 |
| **Model 2** | | | | |
| AF (vs no AF) | 1.05 | 0.52 | 2.18 | 0.885 |
| Age at first TTE (y) | 1.05 | 1.02 | 1.09 | 0.004 |
| Male (vs Female) | 1.00 | 0.54 | 1.88 | 0.994 |
| Time between TTE (y) | 1.26 | 1.12 | 1.42 | <0.001 |
| Hypertension (Yes vs No) | 0.88 | 0.48 | 1.64 | 0.693 |
| Coronary artery disease (Yes vs No) | 1.23 | 0.69 | 2.21 | 0.487 |
| Diabetes (Yes vs No) | 0.55 | 0.28 | 1.05 | 0.077 |
| Previous heart failure (Yes vs No) | 1.00 | 0.56 | 1.77 | 0.987 |

**Table S3:** **Multivariable logistic regression model for predictors of any mitral regurgitation progression.** The table presents odds ratios (ORs) with corresponding 95% confidence intervals (CIs) and *p*-values for all non-echocardiographic covariates. Abbreviations**:** OR = odds ratio; CI = confidence interval; TTE = transthoracic echocardiography; AF = atrial fibrillation.

| **Covariate** | **Odds Ratio** | **Lower 95% CI** | **Upper 95% CI** | **p-value** |
| --- | --- | --- | --- | --- |
| **Model 1** | | | | |
| AF (vs no AF) | 1.02 | 0.42 | 2.87 | 0.972 |
| Age at first TTE (y) | 1.06 | 1.02 | 1.11 | 0.003 |
| Male (vs Female) | 0.88 | 0.47 | 1.72 | 0.706 |
| Time between TTE (y) | 1.20 | 1.07 | 1.36 | 0.003 |
| **Model 2** | | | | |
| AF (vs no AF) | 1.38 | 0.54 | 4.05 | 0.529 |
| Age at first TTE (y) | 1.08 | 1.04 | 1.13 | <0.001 |
| Male (vs Female) | 0.67 | 0.33 | 1.38 | 0.264 |
| Time between TTE (y) | 1.20 | 1.06 | 1.37 | 0.006 |
| Hypertension (Yes vs No) | 1.17 | 0.58 | 2.53 | 0.672 |
| Coronary artery disease (Yes vs No) | 1.08 | 0.55 | 2.11 | 0.817 |
| Diabetes (Yes vs No) | 0.68 | 0.29 | 1.47 | 0.349 |
| Previous heart failure (Yes vs No) | 1.07 | 0.55 | 2.04 | 0.828 |

**Table S4:** **Multivariable logistic regression model for predictors of clinically relevant mitral regurgitation progression.** The table presents odds ratios (ORs) with corresponding 95% confidence intervals (CIs) and *p*-values for all non-echocardiographic covariates. Abbreviations**:** OR = odds ratio; CI = confidence interval; TTE = transthoracic echocardiography; AF = atrial fibrillation.

| **Baseline grade** | **Predicted probability** | **Lower 95% CI** | **Upper 95% CI** |
| --- | --- | --- | --- |
| **No AF** | | | |
| 0 | 0.742 | 0.573 | 0.847 |
| 1 | 0.600 | 0.437 | 0.727 |
| 2 | 0.198 | 0.116 | 0.327 |
| 3 | 0.011 | 0.001 | 0.082 |
| **AF** | | | |
| 0 | 0.751 | 0.632 | 0.835 |
| 1 | 0.611 | 0.499 | 0.702 |
| 2 | 0.206 | 0.159 | 0.276 |
| 3 | 0.012 | 0.002 | 0.078 |

**Table S5:** **Predicted probability of any mitral regurgitation progression by baseline grade and stratified by AF status.** Values represent model-based predicted probabilities averaged over the empirical distribution of all covariates, with corresponding 95% confidence intervals (CIs).

| **Baseline grade** | **Predicted probability** | **Lower 95% CI** | **Upper 95% CI** |
| --- | --- | --- | --- |
| **No AF** | | | |
| 0 | 0.104 | 0.037 | 0.274 |
| 1 | 0.123 | 0.051 | 0.282 |
| 2 | 0.171 | 0.081 | 0.335 |
| 3 | 0.007 | 0.001 | 0.068 |
| **AF** | | | |
| 0 | 0.136 | 0.070 | 0.263 |
| 1 | 0.160 | 0.095 | 0.268 |
| 2 | 0.216 | 0.167 | 0.288 |
| 3 | 0.010 | 0.002 | 0.069 |

**Table S6:** **Predicted probability of clinically relevant mitral regurgitation progression by baseline grade, stratified by AF status.** Values represent model-based predicted probabilities averaged over the empirical distribution of all covariates, with corresponding 95% confidence intervals (CIs).

| **Covariate** | **Odds Ratio** | **Lower 95% CI** | **Upper 95% CI** | **p-value** |
| --- | --- | --- | --- | --- |
| **Model 1** | | | | |
| AF (vs no AF) | 2.22 | 1.15 | 4.54 | 0.023 |
| Age at first TTE (y) | 1.03 | 1.00 | 1.05 | 0.049 |
| Male (vs Female) | 0.93 | 0.57 | 1.50 | 0.755 |
| Time between TTE (y) | 1.08 | 0.99 | 1.18 | 0.081 |
| **Model 2** | | | | |
| AF (vs no AF) | 3.79 | 1.83 | 8.27 | <0.001 |
| Age at first TTE (y) | 1.07 | 1.03 | 1.10 | <0.001 |
| Male (vs Female) | 0.77 | 0.44 | 1.33 | 0.346 |
| Time between TTE (y) | 1.09 | 0.99 | 1.21 | 0.074 |
| Hypertension (Yes vs No) | 0.89 | 0.50 | 1.57 | 0.676 |
| Coronary artery disease (Yes vs No) | 0.94 | 0.56 | 1.59 | 0.826 |
| Diabetes (Yes vs No) | 1.14 | 0.65 | 2.01 | 0.649 |
| Previous heart failure (Yes vs No) | 1.49 | 0.90 | 2.50 | 0.124 |

**Table S7:** **Multivariable logistic regression model for predictors of any tricuspid regurgitation progression.** The table presents odds ratios (ORs) with corresponding 95% confidence intervals (CIs) and *p*-values for all non-echocardiographic covariates. Abbreviations**:** OR = odds ratio; CI = confidence interval; TTE = transthoracic echocardiography; AF = atrial fibrillation.

| **Covariate** | **Odds Ratio** | **Lower 95% CI** | **Upper 95% CI** | **p-value** |
| --- | --- | --- | --- | --- |
| **Model 1** | | | | |
| AF (vs no AF) | 3.66 | 1.22 | 15.89 | 0.041 |
| Age at first TTE (y) | 1.13 | 1.09 | 1.18 | <0.001 |
| Male (vs Female) | 0.76 | 0.42 | 1.39 | 0.358 |
| Time between TTE (y) | 1.23 | 1.01 | 1.39 | <0.001 |
| **Model 2** | | | | |
| AF (vs no AF) | 3.97 | 1.25 | 17.82 | 0.035 |
| Age at first TTE (y) | 1.14 | 1.09 | 1.20 | <0.001 |
| Male (vs Female) | 0.67 | 0.35 | 1.28 | 0.221 |
| Time between TTE (y) | 1.21 | 1.07 | 1.37 | 0.002 |
| Hypertension (Yes vs No) | 1.37 | 0.66 | 3.00 | 0.407 |
| Coronary artery disease (Yes vs No) | 1.07 | 0.57 | 1.97 | 0.834 |
| Diabetes (Yes vs No) | 1.22 | 0.60 | 2.43 | 0.582 |
| Previous heart failure (Yes vs No) | 1.17 | 0.64 | 2.11 | 0.606 |

**Table S8:** **Multivariable logistic regression model for predictors of clinically relevant tricuspid regurgitation progression.** The table presents odds ratios (ORs) with corresponding 95% confidence intervals (CIs) and *p*-values for all non-echocardiographic covariates. Abbreviations**:** OR = odds ratio; CI = confidence interval; TTE = transthoracic echocardiography; AF = atrial fibrillation.

| **Baseline grade** | **Predicted probability** | **Lower 95% CI** | **Upper 95% CI** |
| --- | --- | --- | --- |
| **No AF** | | | |
| 0 | 0.446 | 0.270 | 0.636 |
| 1 | 0.323 | 0.202 | 0.486 |
| 2 | 0.120 | 0.062 | 0.241 |
| 3 | 0.051 | 0.020 | 0.136 |
| **AF** | | | |
| 0 | 0.734 | 0.605 | 0.826 |
| 1 | 0.621 | 0.528 | 0.706 |
| 2 | 0.326 | 0.256 | 0.409 |
| 3 | 0.165 | 0.093 | 0.279 |

**Table S9:** **Predicted probability of any tricuspid regurgitation progression by baseline grade, stratified by AF status.** Values represent model-based predicted probabilities averaged over the empirical distribution of all covariates, with corresponding 95% confidence intervals (CIs).

| **Baseline grade** | **Predicted probability** | **Lower 95% CI** | **Upper 95% CI** |
| --- | --- | --- | --- |
| **No AF** | | | |
| 0 | 0.069 | 0.019 | 0.217 |
| 1 | 0.055 | 0.016 | 0.159 |
| 2 | 0.122 | 0.044 | 0.305 |
| 3 | 0.045 | 0.012 | 0.156 |
| **AF** | | | |
| 0 | 0.203 | 0.118 | 0.330 |
| 1 | 0.170 | 0.112 | 0.257 |
| 2 | 0.312 | 0.254 | 0.396 |
| 3 | 0.143 | 0.085 | 0.248 |

**Table S10:** **Predicted probability of clinically relevant tricuspid regurgitation progression by baseline grade, stratified by AF status.** Values represent model-based predicted probabilities averaged over the empirical distribution of all covariates, with corresponding 95% confidence intervals (CIs).

| **Covariate** | **Beta (ml/m²)** | **Lower 95% CI** | **Upper 95% CI** | **p-value** |
| --- | --- | --- | --- | --- |
| **Model 1** | | | | |
| AF (vs no AF) | -0.27 | -5.44 | 4.90 | 0.918 |
| Age at first TTE (y) | 0.10 | -0.11 | 0.31 | 0.331 |
| Male (vs Female) | -2.88 | -6.71 | 0.95 | 0.140 |
| Time between TTE (y) | 0.97 | 0.28 | 1.66 | 0.006 |
| **Model 2** | | | | |
| AF (vs no AF) | 2.80 | -2.56 | 8.15 | 0.305 |
| Age at first TTE (y) | 0.20 | -0.01 | 0.41 | 0.065 |
| Male (vs Female) | -1.69 | -5.59 | 2.21 | 0.395 |
| Time between TTE (y) | 0.86 | 0.17 | 1.54 | 0.014 |
| Hypertension (Yes vs No) | -0.56 | -4.53 | 3.42 | 0.783 |
| Coronary artery disease (Yes vs No) | -0.98 | -4.7 | 2.75 | 0.605 |
| Diabetes (Yes vs No) | 0.05 | -4.28 | 4.37 | 0.983 |
| Previous heart failure (Yes vs No) | 0.74 | -2.95 | 4.43 | 0.694 |
| Baseline LAVi (mL/m²) | -0.18 | -0.27 | -0.09 | <0.001 |

**Table S11: Multivariable linear regression analysis for change in left atrial volume index.** Regression coefficients (β) with 95% confidence intervals and p-values. Abbreviations: AF = atrial fibrillation; LAVi = left atrial volume index; TTE = transthoracic echocardiography; CI = confidence interval.

| **Covariate** | **Beta (ml/m²)** | **Lower 95% CI** | **Upper 95% CI** | **p-value** |
| --- | --- | --- | --- | --- |
| **Model 1** | | | | |
| AF (vs no AF) | 3.49 | -3.09 | 10.08 | 0.297 |
| Age at first TTE (y) | 0.07 | -0.20 | 0.34 | 0.612 |
| Male (vs Female) | 1.74 | -3.14 | 6.62 | 0.483 |
| Time between TTE (y) | -0.89 | -1.77 | 0.00 | 0.050 |
| **Model 2** | | | | |
| AF (vs no AF) | 5.94 | -0.42 | 12.30 | 0.067 |
| Age at first TTE (y) | -0.03 | -0.30 | 0.24 | 0.824 |
| Male (vs Female) | 3.61 | -1.14 | 8.37 | 0.136 |
| Time between TTE (y) | -0.73 | -1.57 | 0.11 | 0.087 |
| Hypertension (Yes vs No) | -2.92 | -7.73 | 1.89 | 0.234 |
| Coronary artery disease (Yes vs No) | 3.00 | -1.72 | 7.71 | 0.212 |
| Diabetes (Yes vs No) | 3.62 | -1.79 | 9.04 | 0.189 |
| Previous heart failure (Yes vs No) | 1.87 | -2.76 | 6.49 | 0.428 |
| Baseline LVEDVi (mL/m²) | -0.36 | -0.48 | -0.25 | <0.001 |

**Table S12: Multivariable linear regression analysis for change in left ventricular enddiastolic volume index.** Regression coefficients (β) with 95% confidence intervals and p-values. Abbreviations: AF = atrial fibrillation; LVEDVi = left ventricular enddiastolic volume index; TTE = transthoracic echocardiography; CI = confidence interval.

| **Covariate** | **Beta (%)** | **Lower 95% CI** | **Upper 95% CI** | **p-value** |
| --- | --- | --- | --- | --- |
| **Model 1** | | | | |
| AF (vs no AF) | -2.21 | -5.43 | 1.01 | 0.177 |
| Age at first TTE (y) | -0.12 | -0.25 | 0.01 | 0.067 |
| Male (vs Female) | 0.28 | -2.18 | 2.74 | 0.822 |
| Time between TTE (y) | 0.22 | -0.23 | 0.66 | 0.346 |
| **Model 2** | | | | |
| AF (vs no AF) | -3.99 | -6.85 | -1.12 | 0.006 |
| Age at first TTE (y) | -0.10 | -0.21 | 0.02 | 0.092 |
| Male (vs Female) | 0.35 | -1.86 | 2.56 | 0.756 |
| Time between TTE (y) | 0.03 | -0.36 | 0.42 | 0.881 |
| Hypertension (Yes vs No) | 0.43 | -1.85 | 2.72 | 0.710 |
| Coronary artery disease (Yes vs No) | -4.69 | -6.8 | -2.51 | <0.001 |
| Diabetes (Yes vs No) | -0.85 | -3.24 | 0.484 | 0.484 |
| Previous heart failure (Yes vs No) | -1.54 | -3.88 | 0.81 | 0.199 |
| Baseline LVEF (%) | -0.51 | -0.61 | -0.42 | <0.001 |

**Table S13: Multivariable linear regression analysis for change in left ventricular ejection fraction.** Regression coefficients (β) with 95% confidence intervals and p-values. Abbreviations: AF = atrial fibrillation; LVEF = left ventricular ejection fraction; TTE = transthoracic echocardiography; CI = confidence interval.

| **Etiology** | **n** | **Progression (n, %)** | **No progression (n, %)** | **Event rate (95% CI)** |
| --- | --- | --- | --- | --- |
| Primary | 15 | 0 (0.0%) | 15 (100.0%) | 0.0% (0.0–21.8%) |
| Secondary (overall) | 55 | 1 (1.8%) | 54 (98.2%) | 1.8% (0.0–9.7%) |
| Secondary – atrial | 35 | 0 (0.0%) | 35 (100.0%) | 0.0% (0.0–10.0%) |
| Secondary – ventricular | 18 | 1 (5.6%) | 17 (94.4%) | 5.6% (0.1–27.3%) |
| Secondary – mixed | 2 | 0 (0.0%) | 2 (100.0%) | 0.0% (0.0–84.2%) |
| **Total** | **70** | **1 (1.4%)** | **69 (98.6%)** | **1.4% (0.0–7.7%)** |

**Table S14:** **Progression of mitral regurgitation from grade 3 to grade 4 within the AF cohort, stratified by etiology**. Shown are the number of patients with and without progression, as well as event rates with 95% confidence intervals. Abbreviations**:** AF = atrial fibrillation; CI = confidence interval.
